# Supplementary material for: A systematic review of the data, methods and environmental covariates used to map Aedes-borne arbovirus transmission risk
Source: BMC Infect Dis. 2023 Oct 20;23:708. doi: 10.1186/s12879-023-08717-8 (PMC10588093; doi:10.1186/s12879-023-08717-8)
Supplement: Supplementary file 2 — Supplementary Material 2 [file 12879_2023_8717_MOESM2_ESM.docx]

| # | **Question** |
| --- | --- |
| 1 | Does the paper clearly address aims and objectives? |
| 2 | Is the setting and population (e.g. geographical location, time span) clearly defined? |
| 3 | Explicitly describe the origin of input source data, with references |
| 4 | Is the model structure clearly described and appropriate for the research question? (e.g., covariates included) |
| 5 | Is the prediction made for an observation that was not part of the data sample (out-of-sample validation)? |
| 6 | Describe the model performance evaluation method used, with justification |
| 7 | Describe the spatial resolution/scale of risk maps |
| 8 | Are data limitations discussed? |
| 9 | Are the results of the study discussed in context and generalisability considered? |

**Additional file 2. A modified EPIFORGE checklist**
