# Supplementary material for: A systematic review of the data, methods and environmental covariates used to map Aedes-borne arbovirus transmission risk
Source: BMC Infect Dis. 2023 Oct 20;23:708. doi: 10.1186/s12879-023-08717-8 (PMC10588093; doi:10.1186/s12879-023-08717-8)
Supplement: Supplementary file 3 — Supplementary Material 3 [file 12879_2023_8717_MOESM3_ESM.docx]

**Supplementary information**

Supplement to “A systematic review of the data, methods and environmental covariates used to map Aedes-borne arbovirus transmission risk”

**Ah-Young Lim*, Yalda Jafari, Jamie M. Caldwell, Hannah E. Clapham, Katy A. M. Gaythorpe, Laith Hussain-Alkhateeb, Michael A. Johansson, Moritz U. G. Kraemer, Richard J. Maude, Clare P. McCormack, Jane P. Messina, Erin A. Mordecai, Ingrid B. Rabe, Robert C. Reiner Jr, Sadie J. Ryan, Henrik Salje, Jan C. Semenza, Diana P. Rojas, Oliver J. Brady**

*Ahyoung.Lim@lshtm.ac.uk

Contents

[Figure S1. Spatial scale (a) and resolution (b) by study region. 2](#_Toc137633737)

[Figure S2. Time span of data used by disease. 3](#_Toc137633738)

[Figure S3. Summary of lagged covariates used. 4](#_Toc137633739)

[Table S1. Temporal resolution of predictions in reviewed studies. 5](#_Toc137633740)

[Table S2. Number of studies that used robust variable selection procedures. 5](#_Toc137633741)

[Table S3. Modelling methods used in arbovirus risk mapping. 6](#_Toc137633742)

[Table S4. Model performance evaluation metrics used in arbovirus risk mapping. 7](#_Toc137633743)


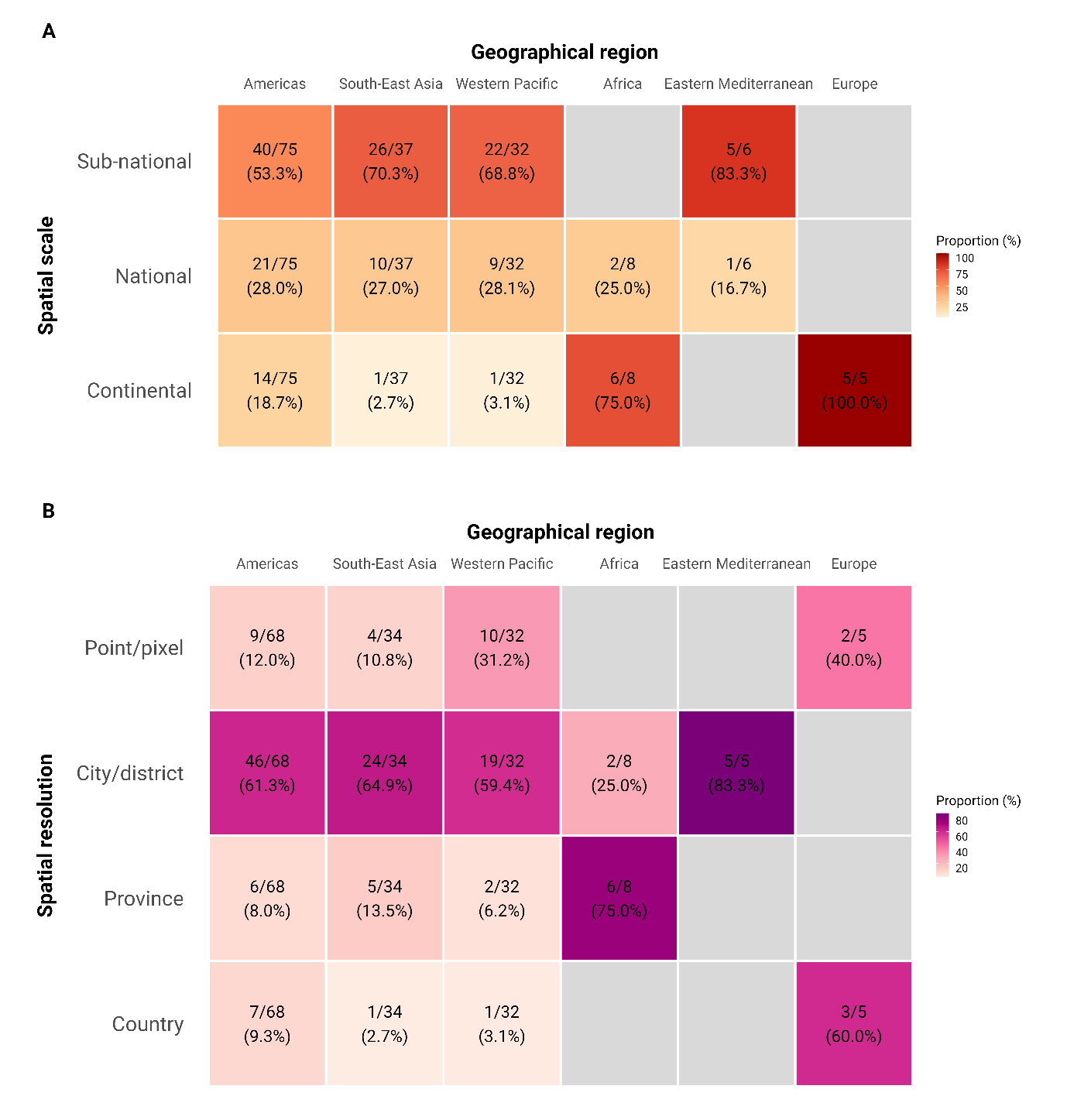


# Figure S1. Spatial scale (a) and resolution (b) by study region.

Each cell represents the number and percentage of studies with the denominators summed vertically.


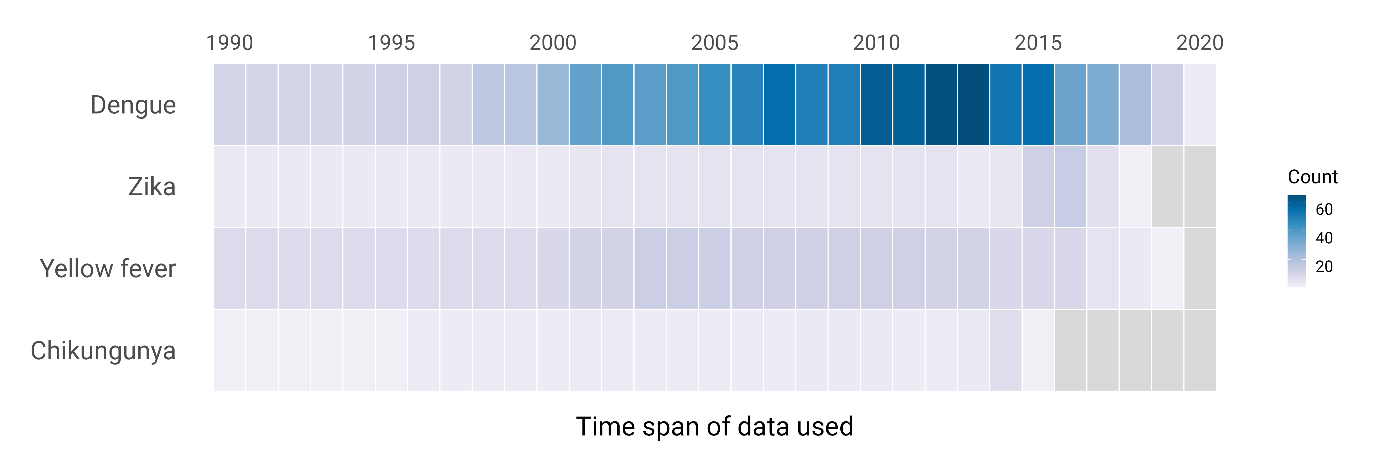


# Figure S2. Time span of data used by disease.


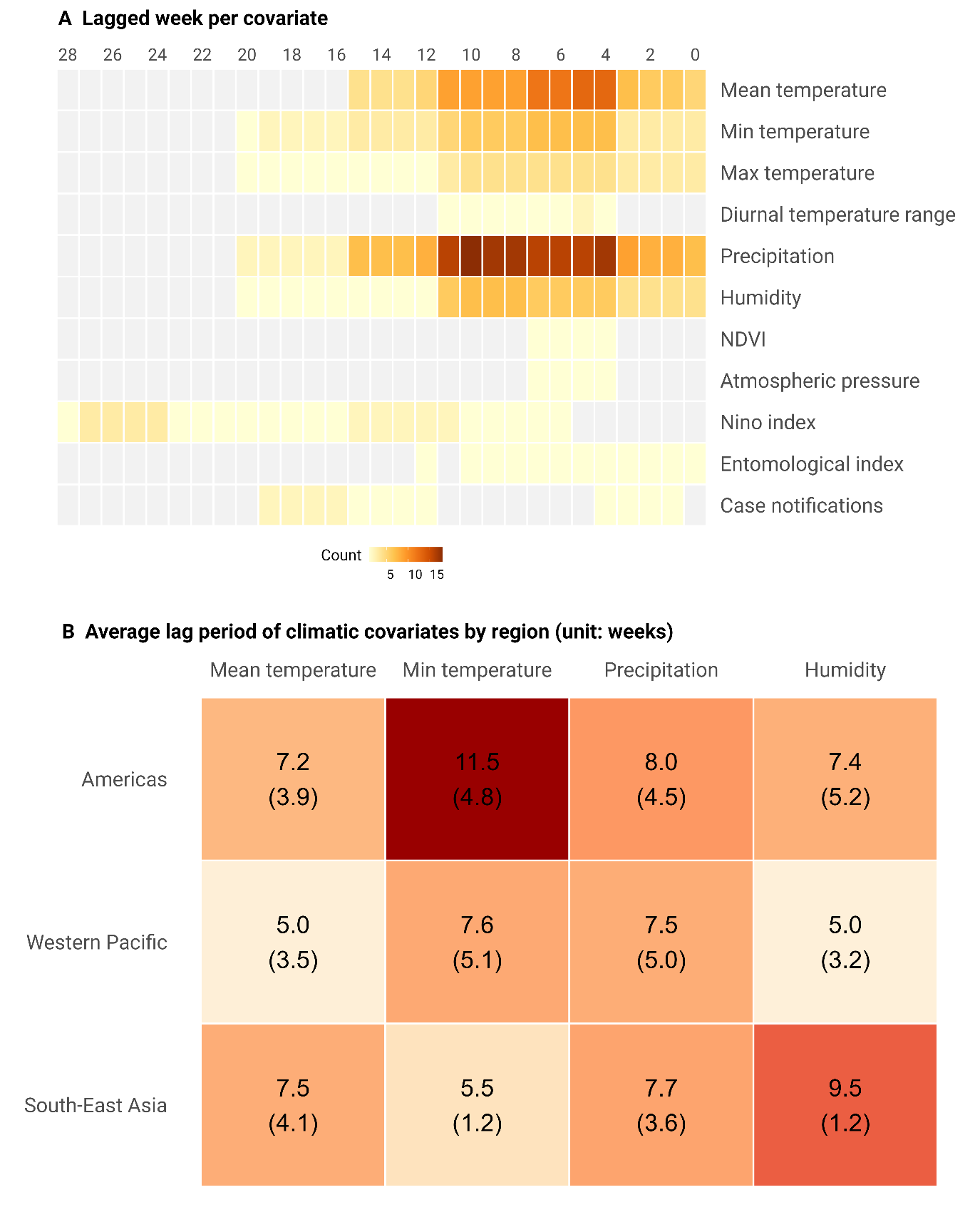


# Figure S3. Summary of lagged covariates used.

(a) lagged week per covariate; (b) average lag period of climatic covariates by region. The numbers represent the mean (standard deviation) of the lag period in weeks.

# Table S1. Temporal resolution of predictions in reviewed studies.

| **Temporal resolution** | **Number of studies** | **Percentage (%)** |
| --- | --- | --- |
| Daily | 2 | 1.1 |
| Weekly | 27 | 15.3 |
| Biweekly | 1 | 0.6 |
| Monthly | 43 | 24.4 |
| Seasonally | 4 | 2.3 |
| Yearly | 12 | 6.8 |
| Aggregated | 86 | 48.9 |
| Not specified | 1 | 0.6 |
| **Grand total** | 176 | 100.0 |

# Table S2. Number and percentage of studies that used robust variable selection procedures.

|  |  | **Collinearity checked** | |  |
| --- | --- | --- | --- | --- |
| **Covariates tested and selected** |  | Yes | No | Total |
|  | Yes | **31 (21.5)** | 36 (25.0) | 67 (46.5) |
|  | No | 16 (11.1) | **61 (42.4)** | 77 (53.5) |
|  | Total | 47 (32.6) | 97 (67.4) | 144 (100.0) |

# Table S3. Modelling methods used in arbovirus risk mapping.

| **Modelling methods** | | **Number of studies** | **Percentage (%)** |
| --- | --- | --- | --- |
| **Statistical mixed effect models** | | **69** | **39.2** |
|  | GLMM | 59 | 33.5 |
|  | GAMM | 5 | 2.8 |
|  | DLNM | 4 | 2.3 |
|  | GLMM and GAMM | 1 | 0.6 |
| **Statistical fixed effect models** | | **39** | **22.2** |
|  | GLM | 18 | 10.2 |
|  | GWR | 11 | 6.3 |
|  | GAM | 9 | 5.1 |
| **Machine learning** | | **48** | **27.3** |
|  | MaxEnt | 10 | 5.7 |
|  | Boosted regression tree | 9 | 5.1 |
|  | Mixed methods | 6 | 3.4 |
|  | Random forest | 6 | 3.4 |
|  | Neural network | 5 | 2.8 |
|  | Other tree based models | 3 | 1.7 |
|  | Others | 7 | 4.0 |
| **Mechanistic models** | | **25** | **14.2** |
|  | Compartmental | 6 | 3.4 |
|  | Metapopulation | 6 | 3.4 |
|  | Network model | 1 | 0.6 |
|  | Others | 12 | 6.8 |

*Some articles are listed more than once.

# Table S4. Model performance evaluation metrics used in arbovirus risk mapping.

| **Model performance metrics** | | **Number of studies** | **Percentage (%)** |
| --- | --- | --- | --- |
| **Correlation metrics** | | **38** | **21.6** |
|  | R-squared | 11.4 | 12 |
|  | Correlation coefficient | 10.2 | 10.4 |
| **Error-based metrics** | | **36** | **20.5** |
|  | Root mean square error (RMSE) | 7.4 | 7.1 |
|  | Mean absolute error (MAE) | 6.3 | 6 |
|  | Mean squared error (MSE) | 1.1 | 1.6 |
|  | Others | 5.7 | 5.5 |
| **Confusion matrix-based metrics** | | **58** | **33.0** |
|  | AUC/ROC | 20.5 | 21.9 |
|  | Kappa statistic | 3.4 | 3.3 |
|  | Accuracy | 1.1 | 1.6 |
|  | Sensitivity and specificity | 1.7 | 1.6 |
|  | Others | 1.7 | 3.3 |
| **Information criteria** | | **82** | **46.6** |
|  | AIC, BIC | 15.9 | 18 |
|  | DIC | 18.2 | 17.5 |
|  | WAIC | 7.4 | 7.1 |
|  | QAIC | 2.3 | 2.2 |
| **Others** | | **34** | **19.3** |
|  | Log likelihood | 3.4 | 3.3 |
|  | Brier score | 1.1 | 1.1 |
|  | Visual comparison | 4.0 | 3.8 |
|  | Others | 8.0 | 10.4 |
| **Not specified** | | **20** | **11.4** |

*Some articles are listed more than once.
